# Supplementary figures and images for: Female sex protects against renal edema, but not lung edema, in mice with partial deletion of the endothelial barrier regulator Tie2 compared to male sex
Source: PLoS One. 2023 Nov 16;18(11):e0293673. doi: 10.1371/journal.pone.0293673 (PMC10653528; doi:10.1371/journal.pone.0293673)

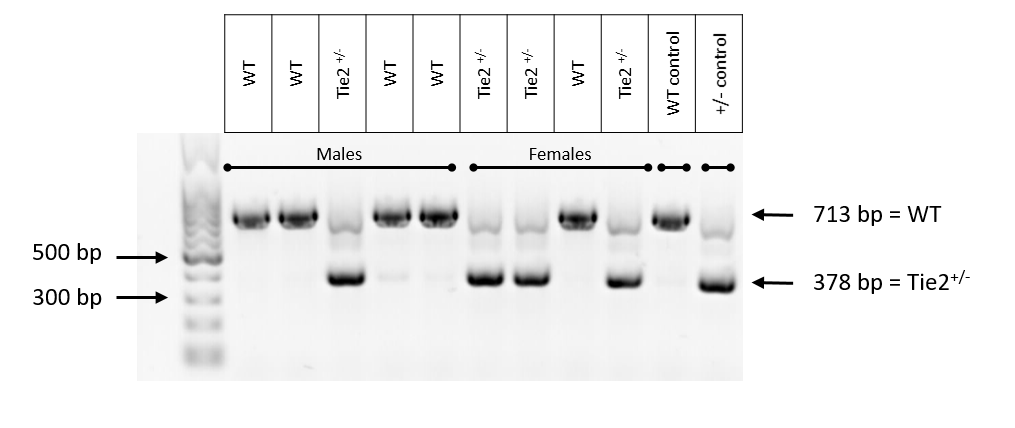

Supplement: S1 Fig — (TIF) [file pone.0293673.s001.tif]
